# Reshoring by Decree? The Effects of Decoupling Europe from Global Value Chains

**DOI:** 10.1007/s10272-022-1087-9

**Published:** 2022-12-16

**Authors:** Alexander Sandkamp

**Affiliations:** Trade Policy, Kiel Institute for the World Economy, Kiellinie 66, 24105 Kiel, Germany

**Keywords:** F11, F13, F17

## Abstract

Overall, decoupling from certain countries may be both politically necessary and economically feasible, at least in the long run. However, a general shift of production back to Europe would be accompanied by significant losses in real income.

Political opposition to ever-increasing globalisation up to the point of calls for deglobalisation seem to be spreading like wildfire. Be it the presidency of Donald Trump, Brexit or China's dual circulation strategy, the past few years have witnessed a politically induced shift towards reduced economic cooperation across countries. Most recently, the two major challenges to globalisation are without a doubt the COVID-19 pandemic and the war in Ukraine. Both have demonstrated the vulnerability of the global economy, thus inducing governments to openly question the benefits of supply chains that span the entire planet.

Three consequences should be noted. First, the pandemic and the war have indeed severely disrupted global production networks. In 2020, medical products such as face masks, protective gloves and ventilators were in short supply. In 2021, many other products including wood, containers and microchips experienced shortages. Now the focus is on energy products, in particular oil and gas. Second, this imbalance between demand and supply has led to a general increase in prices. In the European Union (EU), inflation was already at 2.9% in 2021, i.e. before the war in Ukraine (Eurostat, 2022a). In September 2022, prices increased by 10.9% year-on-year. Third, the disruptions have led companies and governments to question the reliability of global supply chains. This scepticism is partly unjustified, since shortages were also driven by the strong increase in demand for medical products in 2020 as well as the general resurgence in demand that accompanied the global recovery in 2021. EU imports actually increased by 29% in 2021 (Eurostat, 2022b), surpassing the pre-pandemic level. Nevertheless, the impression remains that global supply chains are not as resilient as previously thought.

## Overview of decoupling scenarios

One way to seemingly increase resilience to such bottlenecks in global value chains is to shift production back to the domestic economy. It is argued that this would not only increase resilience, but also reduce dependence on systemic rivals such as China. Based on findings by Felbermayr et al. (2021a, 2022), this article investigates the impact of decoupling the EU from global value chains on trade and welfare (i.e. real income). In particular, four specific scenarios are presented.

The first scenario investigates a unilateral decoupling by the EU from the rest of the world with the aim of reducing imports and shifting production back to EU countries. The second scenario investigates a mutual decoupling, meaning the explicit restriction not only of EU imports but also exports.[1] Against the backdrop of increased dependence on China, the third scenario simulates the impact of a mutual decoupling of the EU from China only. Finally, the fourth scenario models a mutual decoupling of the EU, the US and their allies from Russia.

## Dependencies on non-European suppliers

Before investigating the potential impact of decoupling the EU from global value chains, it is worth taking a look at an example that illustrates the current dependence of the EU on global value chains. Germany, Europe's largest economy, sourced 92% of its intermediate products from Europe in 2018 (OECD, 2021). Relocating the remaining 8% may thus seem feasible. In 2018, only 1.3% of total direct intermediate products (6% of imported intermediates) came from China. However, these aggregates mask significant heterogeneity across products. For example, 80% of laptops and 70% of mobile phones imported into Germany originated from China in 2021. In a recent survey among German companies, 46% of respondents said that they were currently sourcing important intermediate products from China (Baur and Flach, 2022). Decoupling from China, let alone the world, may therefore be difficult – at least in the short run.

### Methodology

The four scenarios are simulated using the Kiel Institute Trade Policy Evaluation (KITE) model. It is a general equilibrium trade model that simulates trade flows between 141 countries

**Alexander Sandkamp**, Kiel University (CAU); and Kiel Institute for the World Economy (IfW), Germany.

1 It does not matter whether the EU imposes barriers on exports to third countries or whether these countries impose barriers on their imports from the EU. Within the model, both actions have the same effect on trade and real income.

Table 1
**Trade effects following EU decoupling**

| EU | Change in real imports (in %) | Change in real exports (in %) |
|---|---|---|
| Scenario I (Unilateral decoupling) | -62.5 | -54.2 |
| Scenario II (Trade war) | -84.1 | -87.0 |

Note: Without China (effects similar). Excluding oil and gas.

Source: Felbermayr et al. (2021a).

Table 2
**Change in real income following EU decoupling**

| EU | Change in real income (in %) | Income change (in bn euro) |
|---|---|---|
| Scenario I (Unilateral decoupling) | -3.5 | -584.4 |
| Scenario II (Trade war) | -5.3 | -873.1 |

Note: Change in income based on GDP in 2019. EU28 including the UK. Excluding oil and gas.

Source: Felbermayr et al. (2021a).

in 65 sectors and covers 98% of global economic activity. The model is calibrated using the latest version of the Global Trade Analysis Project (GTAP; Aguiar et al., 2019) database.[2] Within the model, a shift in production back to the domestic economy is modelled by a doubling of non-tariff barriers (NTBs). These incorporate a plethora of different instruments including import controls, state aid, public procurement and trade defence instruments that have been shown to significantly reduce trade flows (Bratt, 2017; Kinzius et al., 2019). NTBs are doubled because this strongly reduces bilateral trade flows without completely eliminating them (no full shift to autarky). Crucially, the model allows investigating the impact of such a change in trade barriers on trade and real income.

## Simulation results

In a first step, Table 1 shows how a doubling of EU trade barriers vis-à-vis non-EU countries impacts EU imports and exports in the model. Doubling import barriers reduces EU imports from third countries by 62.5% (Scenario I). Raising import barriers shifts production back to Europe. However, this means that EU producers have to forego the benefits from the international division of labour and specialisation as the production of intermediate products is relocated to Europe. Instead of being imported from the cheapest source country, intermediates are now being produced at home at higher cost. Prices of remaining imports also increase due to the imposed trade barriers. In addition, the euro experiences a real appreciation as EU demand for non-EU products falls (in the model, this appreciation is achieved through price adjustments). All this weakens the competitiveness of European exporters, resulting in a 54.2% fall in exports, almost as large as the decline in imports. In a trade war scenario (explicit barriers on imports as well as exports), trade would fall even more strongly.

## Shifting production back to Europe

The effects of such general reshoring of European production on real income are illustrated in Table 2. A unilateral imposition of trade barriers on EU imports would reduce real income by 3.5% (Scenario I). This is a permanent effect and implies that income in the EU would be permanently 3.5% lower than in a world without additional barriers. In terms of 2019 GDP, this amounts to foregone income of €584.4 billion every year. A mutual decoupling (Scenario II) would increase this loss to 5.3%.

The aggregate effects presented in the table mask significant heterogeneity across EU member states. For example, Germany as a very open economy loses more from a trade war (-6.9% or €236.7 billion). Small open economies such as Belgium, Malta and Ireland are hit hardest by a trade war between the EU and its trading partners. Welfare in the EU's trading partners declines, too (on average by 1.5% in Scenario II). Shifting a large part of production back to the EU is thus possible in principle, but would involve considerable costs for both the EU as well as its partners.

## Decoupling the EU from China and Russia

In view of recent geopolitical developments, a strategic decoupling of the EU from specific countries may be more plausible than a general move back towards autarky. Following the Russian invasion of Ukraine on 24 February 2022, EU trade with Russia has declined significantly. In August 2022, EU exports to (imports from) Russia were 46% (27%) lower than in February (Eurostat, 2022b).[3] A decoupling from Russia is thus already in progress (European Commission, 2022a, 2022b). In addition, decoupling from China may also seem increasingly likely as the EU worries about a one-sided dependence on a country that is on "a mission to establish its dominance in East Asia and its influence globally" (European Commission, 2022c) and also keeps close diplomatic and economic ties with Russia. Scenarios III and IV therefore analyse the impact of a decoupling of the EU from China and Russia, respectively.

2 For a detailed description of the model, please see Felbermayr et al. (2022).

3 Compared to August 2021, EU imports from Russia actually increased by 6% in August 2022. This is driven by the strong increase in the price of energy products.

Table 3
**Change in exports and real income following decoupling**

| | Change in bilateral exports (in %) | | Change in real income (in %) | | |
|---|---|---|---|---|---|
| | EU | China | EU | Germany | China |
| Scenario III (Decoupling EU – China) | -97.7 | -96.2 | -0.8 | -1.0 | -0.9 |
| | EU, US, allies | Russia | EU, US, allies | Germany | Russia |
| Scenario IV (Decoupling EU, US, allies – Russia) | -97.7 | -96.4 | -0.2 | -0.4 | -9.7 |

Notes: EU27 excluding UK. The country group allies includes Albania, Australia, Canada, Iceland, Japan, New Zealand, Norway, the Philippines, South Korea, Taiwan, Turkey and the United Kingdom. Including oil and gas.

Source: Felbermayr et al. (2022).

Simulation results are reported in Table 3. Doubling import and export barriers between the EU and China almost completely eliminates bilateral trade (Scenario III). EU exports to China decline by 97.7%, while Chinese exports to the EU fall by 96.2%. Intuitively, it is easier for the EU to decouple from one country – even one as large as China – than from many countries. Consequently, the fall in bilateral trade is stronger than in Scenario II as some trade is now diverted to other countries. Real income declines by 0.8% in the EU and by 0.9% in China in this scenario.

Decoupling from Russia is modelled in Scenario IV. In line with recent developments, this scenario assumes joint action by the EU, the US and their allies. The impact on bilateral trade between the EU, the US and their allies on one side and Russia on the other is comparable to that between the EU and China in Scenario III. However, the effect on income is very different. The EU, the US and their allies lose 0.2% on average. Once again, there is significant heterogeneity within the EU, with Eastern European economies suffering most from a decoupling from Russia. Germany, Europe's largest economy, loses 0.4% when decoupling from Russia, compared to 1% when decoupling from China. On the other hand, Russia loses much more from decoupling (9.7% in Scenario IV) than China (0.9% in Scenario III).

These varying effects can be explained by differences in relative economic size. To give an example, 22% of EU imports came from China in 2021, while Russia was only responsible for 7% of EU imports (UN, 2022). It is thus not surprising that the EU loses less from a trade war with Russia than from one with China. On the other hand, the EU, the US and their allies are more important for Russia than just the EU is for China, which is in itself a larger economy than Russia. Consequently, Russia suffers more from a trade war than China. Finally, given that the EU alone accounted for 37% of Russian trade in 2020 compared to a Russian share of 5.8% in EU trade in 2021 (European Commission, 2022d), it is clear that Russia loses more from a trade war than the EU, the US and their allies. This result has important policy implications, as it implies that sanctions will be more effective, the more countries implement them (a finding also put forward by Chowdhry et al., 2022).

The relatively small welfare losses for the EU, the US and their allies may seem surprising at first glance. However, all simulated effects are long-run effects that occur once a new equilibrium has been reached. Specifically, in the case of decoupling from Russia, this means that new gas pipelines and liquefied natural gas terminals have been built and the energy transition has been completed. In the short run, the negative effects are likely to be much stronger (0.5% to 3% for Germany; Bachmann et al., 2022).

## Implications for resilience

The model results provided by Felbermayr et al. (2021a, 2022) have demonstrated that shifting production back to the EU comes at the cost of lower living standards. Besides political considerations, decoupling may still be worthwhile if it increases the resilience of the European economy. Surely, a return to autarky would insulate the EU from shocks in third countries, such as pandemic lockdowns in China or Russian gas export bans. However, it would make the EU more vulnerable to shocks within Europe. These might be less likely, but nevertheless remain possible, as recent floods in Germany and the forced shutdown of nuclear power plants in France have painfully demonstrated. In such cases, international trade traditionally serves as a type of insurance, as urgently needed products can be imported in the event of a crisis. A return to autarky would deprive the EU of such possibilities. The same is true if global shocks occur at slightly different times in different countries. For example, at the time most European countries entered into lockdown in 2022, China was already starting to emerge from its lockdown (albeit only temporarily).

## Alternatives to full decoupling

Bilateral decoupling – as in the case of Russia – may sometimes be politically unavoidable. However, instead of a general reshoring of production, a superior strategy to reduce dependence is diversification. Sourcing intermediate products from different regions would strengthen the resilience of the EU economy while at the same time preserve the advantages of the gains from trade. This diversification can be explicitly facilitated by the European Commission through increasing international cooperation. Specifically, trade agreements can lower trade barriers, for example by mutual recognition of standards, and thus make it easier for European companies to increase the number of source countries. Similarly, investment agreements can reduce risks and thus encourage investment in new production sites abroad. Moreover, the funds earmarked for international investment projects in the context of the EU's Global Gateway initiative (European Commission, 2022e) can be used to invest in energy or raw material production projects. This would benefit people in the countries concerned while at the same time ensuring supply of critical raw materials and energy for Europe.

In addition to actively encouraging diversification, the EU should carefully evaluate potential conflicts of interest with other policy objectives. For example, the European Commission's proposed EU due diligence legislation would require European companies to ensure compliance with certain human and environmental rights standards along the entire supply chain (European Commission, 2022f). As argued by Felbermayr and Sandkamp (2022), such legislation would increase firms' cost per supplier relationship, as EU companies need to control each supplier (as well as each supplier's suppliers) with respect to compliance. These explicit costs, as well as the implicit costs of the risk of being fined for failing to uncover violations of human and environmental rights, incentivise firms to consolidate their supply chains by reducing the number of suppliers (even those that do not violate human rights). The result would be a concentration rather than a diversification of suppliers and thus reduced resilience. This does not mean, of course, that human rights should rank behind supply chain security. However, all legislation has to be carefully evaluated with respect to its potential to hinder increased resilience of supply chains and adjusted where necessary.[4]

Beyond diversification, additional ways to increase resilience include improved warehousing and strategic reserves at the EU level (Felbermayr et al., 2020). Improved recycling systems are particularly important, as the production of certain raw materials can never be shifted to the EU, because they simply do not exist on European soil. At the same time, recycling can contribute to more sustainability and reduced carbon emissions. Finally, new technologies such as 3D printing might actually make certain comprehensive manufacturing processes obsolete and would thus contribute to shortening supply chains and therefore increasing their resilience.

## Conclusion

Overall, decoupling from certain countries may be both politically necessary and economically feasible, at least in the long run. However, a general shift of production back to Europe would be accompanied by significant losses in real income. In addition, while reducing dependence from third countries, it would make the EU more vulnerable to local shocks. Instead of decoupling, the EU should therefore pursue an active policy of increased international cooperation in order to diversify its suppliers and thus increase resilience of its economy. This should be accompanied by initiatives that encourage firms to find technical solutions to supply chain disruptions such as improved recycling techniques and 3D printing. If done correctly, Europe can emerge from the current crises stronger and more open, thus securing prosperity for both its own citizens as well as its partners.

4 For alternatives to the proposed EU due diligence legislation that would not incentivise firms to consolidate supply chains, see Felbermayr et al. (2021b).
